# Sexual dysfunction and quality of life in cervical and endometrial cancer patients before and after low-dose-rate brachytherapy: a cohort study

**DOI:** 10.3389/fmed.2025.1584141

**Published:** 2025-04-28

**Authors:** Celia B. González-Alcorta, Adelina Alcorta-Garza, Daneli Ruiz-Sánchez, Blanca Angélica Soto-Martínez, Fernando Alcorta-Núñez, Itzel Lidey Galaviz-Reynoso, Paola A. López-Sierra, Juan Francisco González-Guerrero, Oscar Vidal-Gutiérrez

**Affiliations:** ^1^Oncology Service, Area of Radiooncology, University Center Against Cancer, University Hospital "José E. González", Universidad Autónoma de Nuevo León, Monterrey, Mexico; ^2^Coordination of Psycho-Oncology and Liaison between Medicine and Palliative Care, University Center Against Cancer, University Hospital "José E. González", Universidad Autónoma de Nuevo León, Monterrey, Mexico; ^3^Oncology Service, University Center Against Cancer, University Hospital "José E. González", Universidad Autónoma de Nuevo León, Monterrey, Mexico; ^4^Oncology Service, Area of Medical Oncology and Radiooncology, University Center Against Cancer, University Hospital "José E. González", Universidad Autónoma de Nuevo León, Monterrey, Mexico; ^5^Oncology Service, Area of Oncologic Surgery, University Center Against Cancer, University Hospital "José E. González", Universidad Autónoma de Nuevo León, Monterrey, Mexico

**Keywords:** sexual dysfunction, quality of life, low-dose-rate, brachytherapy, cervical and endometrial cancer

## Abstract

**Background:**

Research on low-dose-rate (LDR) brachytherapy for gynecological cancer primarily examines treatment toxicity while overlooking aspects such as sexual desire, arousal, orgasm, satisfaction, and overall quality of life. We assessed sexual function and quality of life in patients with cervical and endometrial cancer before and after LDR brachytherapy, identifying factors related to sexual dysfunction and good quality of life 3–6 months after brachytherapy.

**Materials and methods:**

We prospectively followed a cohort of patients with a histopathological diagnosis of cervical and endometrial cancer who were treated with LDR intracavitary brachytherapy (*n* = 139). The SyDSF-AP, FACT-G, PHQ-9, and PHQ-15 scales were collected using a self-administered questionnaire before and 3–6 months after treatment. The analysis included estimating incidence rates and conducting a binary multiple logistic regression.

**Results:**

Sexual dysfunction was observed in 14.4% of individuals, with 30% already affected at baseline. Higher education was associated with a decreased likelihood of developing or maintaining sexual dysfunction (OR, 0.10; 95% CI, 0.01–0.97). Physical wellbeing improved after treatment, with scores increasing from 69.3 to 78.7 (*p* < 0.001; effect size = 0.34). The presence of moderate-to-severe somatic symptoms, major depression, and sexual dysfunction reduced the likelihood of starting or maintaining a good quality of life.

**Conclusion:**

Over 10% of patients experienced sexual dysfunction, with physical wellbeing being the only area that showed improvement after treatment. Research in this area enhances awareness and understanding of how healthcare providers can better support sexual and health-related wellbeing.

## Introduction

There are five main types of gynecological cancer: cervix, uterine (usually endometrial), ovarian, vaginal, and vulvar. The Global Cancer Observatory reported that cervical cancer was the fifth most common malignant disorder in 2022, with an incidence rate of 14.1%. Cervical cancer is the third most common cancer, and uterine cancer is fifth, with rates of 13.2% in Mexico and 22.5% in the United States. Cervical cancer is the leading cause of gynecologic cancer deaths worldwide, especially in low- and middle-income countries. Mexico has the highest mortality rate among gynecological cancers at 6.2% (1). It is standard for cervical cancer patients to receive brachytherapy after external beam radiation therapy. For endometrial cancer patients, it is used post-hysterectomy to target any remaining cancer cells. Additionally, patients with uterine cancer who cannot undergo surgery may also receive brachytherapy (2–4). Brachytherapy may cause early side effects such as pain, swelling, and vaginal bleeding (5, 6). Over time, it may affect the pelvic floor muscles, leading to urinary and anal incontinence (7, 8). Radiotherapy can damage the vaginal structure, resulting in shortened length, reduced lubrication, and decreased elasticity. As a result, patients may experience stenosis, dryness, and dyspareunia (9–11). Survivors of gynecological cancer frequently face psychological challenges, such as reduced libido, body image changes, and anxiety about sexual performance. They may also struggle with maintaining previous sexual roles, feeling emotionally distant from their partner, and concerns about their partner’s sexual interest after treatment (12, 13). All these physical, psychological, and social effects may significantly impact sexual and health-related quality of life.

Brachytherapy can involve a low-dose-rate (LDR) or a high-dose-rate (HDR) of radiation (14). The primary distinctions are the speed and intensity of radiation delivery; HDR provides a rapid and intense dose, whereas LDR offers a slower and more prolonged dose. The choice depends on the type and location of the cancer, the patient’s health, and access to specialized equipment. A pulsed dose rate (PDR) is another type of brachytherapy that delivers continuous pulses over several days, each lasting a few minutes per hour. It combines the physical benefits of HDR treatment with the radiobiological advantages of LDR therapy (15, 16). Its primary disadvantage is the need for a dedicated hospital room with a remote after-loading system. Over time, HDR has replaced LDR (17). However, access to HDR brachytherapy in Latin America and Mexico involves investing in healthcare infrastructure, improving training programs, and addressing healthcare disparities (18). Some studies have explored how HDR brachytherapy affects sexual function and quality of life in gynecological cancer patients (12, 19–21). In contrast, studies on LDR brachytherapy have primarily examined treatment toxicity, such as vaginal mucosal changes, vaginal stenosis, and pain, without addressing sexual desire, arousal, orgasm, satisfaction, or quality of life (22–25). Understanding the impact of LDR brachytherapy on sexual function and quality of life can help guide healthcare policies, funding, and education to address these critical issues for cancer survivors in regions operating LDR units.

This study aimed to evaluate sexual function and quality of life in cervical and endometrial cancer patients before and after LDR brachytherapy. We also identified factors related to sexual dysfunction and good quality of life 3–6 months post-treatment.

## Materials and methods

**Figure 1:**
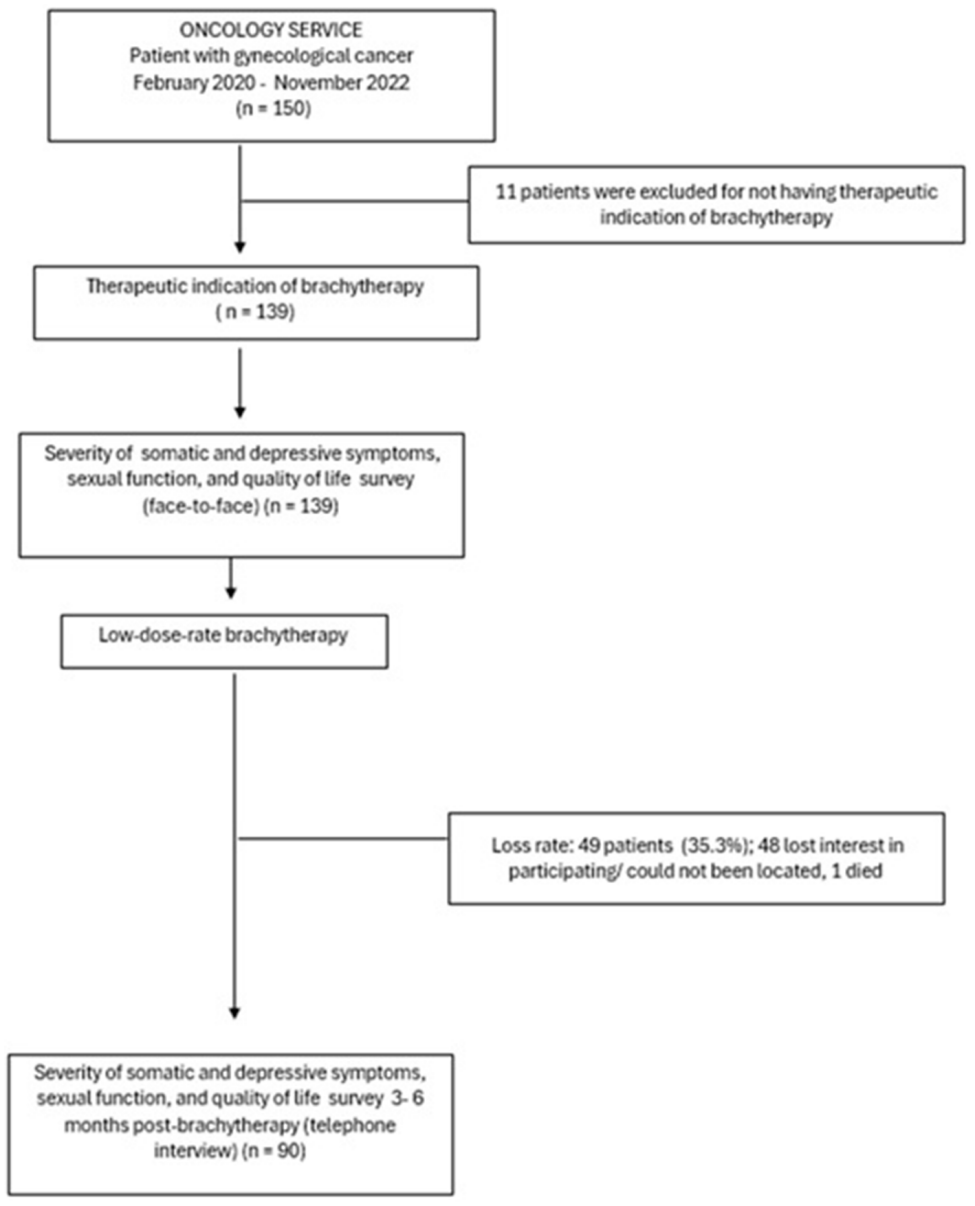
Study design.

We prospectively followed a cohort of patients with a histopathological diagnosis of cervical and endometrial cancer treated with LDR intracavitary brachytherapy between February 2020 and November 2022. Participants had to be at least 18 years old, without neurological or cognitive disorders, and have a confirmed diagnosis of cervical or endometrial cancer. They were selected consecutively from the oncology outpatient clinic of a tertiary public teaching hospital in Monterrey, Mexico (Figure 1). The loss rate was 35.3%. Responders and non-responders showed no significant differences in terms of age, education, marital status, occupation, origin, disease stage, surgery, external radiotherapy, chemotherapy, or brachytherapy sessions (*p* > 0.05). The sample size of 97 was determined based on an expected 50% incidence of sexual dysfunction (SyDSF-AP ≥ 2) 3–6 months after brachytherapy, with a 10% margin of error and 95% confidence level.

### Brachytherapy parameters

The brachytherapy procedure adhered to institutional standards, and its delivery was homogeneous over time. Patients were hospitalized for the entire duration of the procedure. After thorough asepsis, a urinary catheter was inserted into the bladder and secured for physiological filling. Brachytherapy consisted of administering 20 to 60 Gy 5 mm below the vaginal surface using LDR cesium-137 sources. The dose rate ranged from 0.4 to 0.6 Gy/h (10–15 Gy/day). X-rays were used to estimate the doses according to the International Commission on Radiation Units and Measurements points, ensuring precise and effective radiation application. A Siemens Somatom computed tomography scanner from Siemens Healthineers was utilized for imaging evaluation during the treatment. Applicators were removed without anesthesia, and the sources were withdrawn after the required time. In patients receiving surgical treatment, chemotherapy, or external radiotherapy, brachytherapy should be administered at least 1 month after surgery or after 50% of the chemotherapy or external radiotherapy sessions have been completed. All patients were evaluated at 1-month post-treatment and between 3 and 6 months after therapy.

### Standard protocols for cervical and endometrial cancer

The standard treatment for early-stage cervical cancer typically involves a surgical approach. If adverse characteristics are present, treatment may include chemotherapy (5 cycles of cisplatin) or external beam radiation (25 daily fractions), followed by two LDR treatments of 20 Gy to the mucosa. Advanced cervical cancer cases are treated with chemotherapy (5 cycles of cisplatin) and external beam radiation (25 daily fractions), followed by two LDR of 20 Gy to the mucosa. Three patients with stage IVB cervical cancer received LDR brachytherapy as a palliative measure after undergoing chemotherapy and presenting with active bleeding and pain. The standard treatment for early-stage endometrial cancer includes surgery, with or without LDR brachytherapy, depending on individual risk factors. Advanced cases are treated with surgery, chemotherapy (5 cycles of cisplatin), external beam radiation (25 daily fractions), and LDR (1–3 applications to the vaginal cuff). Unresectable cases are treated with chemotherapy (5 cycles of cisplatin) and external beam radiation (25 daily fractions), with or without LDR treatments or chemotherapy.

### Study variables

#### Sexual function

It was measured using the Health and Feminine Sexual Dysfunction in Primary Care (SyDSF-AP) scale developed and validated in Spanish by Casas Aranda et al. (26). Sexual function was assessed based on the experience of symptoms in the past 3 months. The sexual function domain encompassed nine items regarding sexual satisfaction, sexual desire, sexual enjoyment, sexual arousal, dyspareunia, orgasm, sexual difficulties with a partner, the ability to live without sex, and feelings of depression or nervousness because of sexual problems (Cronbach’s alpha before and after brachytherapy = 0.56 and 0.60, respectively). The SyDSF-AP scale responses were on a 6-point Likert scale ranging from 0 (never) to 5 (always). The positively phrased questions were reversed so that a higher score indicated poor sexual function. The responses were averaged and categorized into the presence or absence of sexual dysfunction based on a cutoff point of ≥ 2 (equivalent to sometimes, often, almost always, and always). The classification of changes in status between the baseline measurement (before brachytherapy) and the second measurement taken 3–6 months after brachytherapy is shown in Supplementary file 1.

#### Quality of life

It was measured using the Functional Assessment of Cancer Therapy - General Scale (FACT-G) developed in 1993 for evaluating patients receiving cancer treatment (27) and validated in Spanish (28). It assessed the quality of life over the last 7 days and consisted of four dimensions: (a) physical wellbeing (e.g., “I lack energy”), with seven items (Cronbach’s alpha before and after brachytherapy = 0.86 and 0.82, respectively), (b) family and social wellbeing (e.g., “I receive emotional support from part of my family”), with seven items (Cronbach’s alpha before and after brachytherapy = 0.77 and 0.72, respectively), (c) emotional wellbeing (e.g., “I feel sad”), with six items (Cronbach’s alpha before and after brachytherapy = 0.74 and 0.71, respectively), and (d) functional wellbeing (e.g., “I sleep well”), with seven items (Cronbach’s alpha before and after brachytherapy = 0.81 and 0.78, respectively). FACT-G scale responses were provided on a 5-point Likert scale ranging from 0 (not at all) to 4 (very much). Negatively phrased questions were reversed so that a higher score indicated a better quality of life. The score was then transformed to a 0–100 scale, and the responses were averaged and categorized as follows: poor (0–25, equivalent to not at all and a little bit), fair (26–74, equivalent to somewhat), and good quality of life (75–100, equivalent to quite a bit and very much). The classification of the change in status between the baseline measurement (before brachytherapy) and the second measurement taken 3–6 months after brachytherapy is shown in Supplementary file 1.

#### Somatic and depressive symptom severity

This was assessed using two patient health questionnaires: the PHQ-15 (29) and the PHQ-9 (30). The PHQ-15 assessed physical problems that may have bothered the patient over the past 4 weeks, whereas the PHQ-9 evaluated depressive symptoms experienced in the previous 2 weeks. Both were available in Spanish (31, 32). The PHQ-15 consisted of 15 items, for example, “I have had stomach pain” (Cronbach’s alpha before and after brachytherapy = 0.85 and 0.83, respectively), with responses on a 3-point Likert scale ranging from 0 (not bothered at all) to 2 (bothered a lot). A higher score indicated a greater severity of somatic symptoms. The sum of the responses was categorized into minimal-mild (0–9) and moderate–severe (≥10). The PHQ-9 consisted of nine items, such as “I feel little interest or pleasure in doing things” (Cronbach’s alpha before and after brachytherapy = 0.82 and 0.73, respectively), with responses on a 4-point Likert scale ranging from 0 (never) to 3 (almost every day). A higher score indicated greater severity of depressive symptoms. The sum of the responses was categorized, with a score of ≥ 9 indicating major depression. This cutoff point was chosen because it demonstrated a sensitivity of 95% (29). The classification of the change in status between the baseline measurement (before brachytherapy) and the second measurement taken 3–6 months after brachytherapy is shown in Supplementary file 1.

#### Sociodemographic

Age, schooling, marital status, occupation, origin, and parity.

#### Procedures

Sexual function, quality of life, somatic and depressive symptom severity, and sociodemographic information were collected through a self-administered questionnaire before LDR-brachytherapy (basal measurement). At 3–6 months after brachytherapy, data were collected via telephone (second measurement). Medical files provided information on the patient’s history of diabetes, hypertension, and other health conditions. Brachytherapy characteristics (technique and number of sessions) and other treatments used, including surgery, external beam radiation, and chemotherapy, were recorded and analyzed. Additionally, data were collected regarding side effects and complications (gastrointestinal, genitourinary, fistula, colostomy, and nephrostomy), as well as the response to oncologic treatment (recurrence, progression, or persistence of cancer). The classification of disease stage followed the International Federation of Gynecology and Obstetrics.

#### Statistical analysis

Descriptive statistics, including means and standard deviations, were used for continuous variables, while frequency distributions were applied for categorical variables. The incidence of sexual dysfunction, good quality of life, moderate–severe somatic symptoms, and major depression was estimated based on the change from absence to presence (Supplementary file 1). The Wilcoxon signed-rank test for two related samples was used to compare the mean scores of the quality-of-life domains and the mean number of bothersome somatic and depressive symptoms. The effect size was estimated when the difference in the quality-of-life domain’s pre- and post-measurements was significant. Binary multiple logistic regression was used to examine risk factors for sexual dysfunction (dependent variable coded yes vs. no) and good quality of life (dependent variable coded yes vs. no) after LDR brachytherapy. In both models, age, education level, major depression, moderate–severe somatic symptoms, comorbidities, and side effects/complications were treated as independent variables. Disease stage and other treatments were used to serve as control variables. Odds ratios (OR) and 95% confidence intervals (CI) were estimated.

## Results

The mean age of the study population was 46.2 ± 12.7 years, and the mean number of pregnancies was 3.4 ± 1.7. The majority of participants were married or living with a partner, had a low level of education, were homemakers, and were primarily from the Northeastern states of Mexico, specifically Nuevo León and Tamaulipas. One in five patients had diabetes or hypertension, and more than 40% of the participants experienced gastrointestinal side effects (Table 1).

**Table 1:** Sociodemographic profile and intercurrent diseases (*n* = 139).

Characteristic	Frequency
	*n*	%
Married or living with a partner	89	65.0
Schooling		
Elementary	48	34.5
Secondary	51	36.7
High school	25	18.0
Professional/postgraduate	15	10.8
Employment status		
Housewife	83	59.7
Employed	41	29.5
Unemployed/retired	15	10.8
Origin		
Nuevo Leon	50	36.0
Tamaulipas	54	38.8
Other	35	25.1
Intercurrent diseases		
Any comorbidity	43	30.9
Diabetes	28	20.1
Hypertension	31	22.3
Other (hypothyroidism, psoriasis, arthritis)	7	5.0
Side effects/complications (any)	73	52.5
Side effects/complications type		
Gastrointestinal	61	43.9
Genitourinary	25	18.0
Fistula	31	22.3
Colostomy	21	15.1
Nephrostomy	4	2.9

The disease stages were as follows: 20.4% stage IA or IB, 21.9% stage IIA or IIB, 10.9% stage IIIA or IIIB, 23.4% stage IIIC, and 23.4% stage IVA or IVB. The number of brachytherapy sessions was 2 for 82.7%, 1 for 15.1%, and 3 for 2.2%. The Fletcher technique predominated (61.8%), followed by colpostatos (33.1%) and mini-pelvis (5.1%). The frequency of other treatments received was surgery (36%), chemotherapy (71.9%), and external beam radiation (95.0%). The rates of cancer recurrence, progression, and persistence were 2.9, 5.8, and 10.8%, respectively. The mean number of bothersome somatic symptoms at 3–6 months was lower than before brachytherapy (8.3 ± 5.4 vs. 6.4 ± 4.2, *p* < 0.001). The mean number of depressive symptoms also decreased (7.2 ± 6.0 vs. 5.3 ± 5.1, *p* < 0.01). The incidence of moderate–severe somatic symptoms and major depression was 6.7%.

### Sexual function

**Figure 2:**
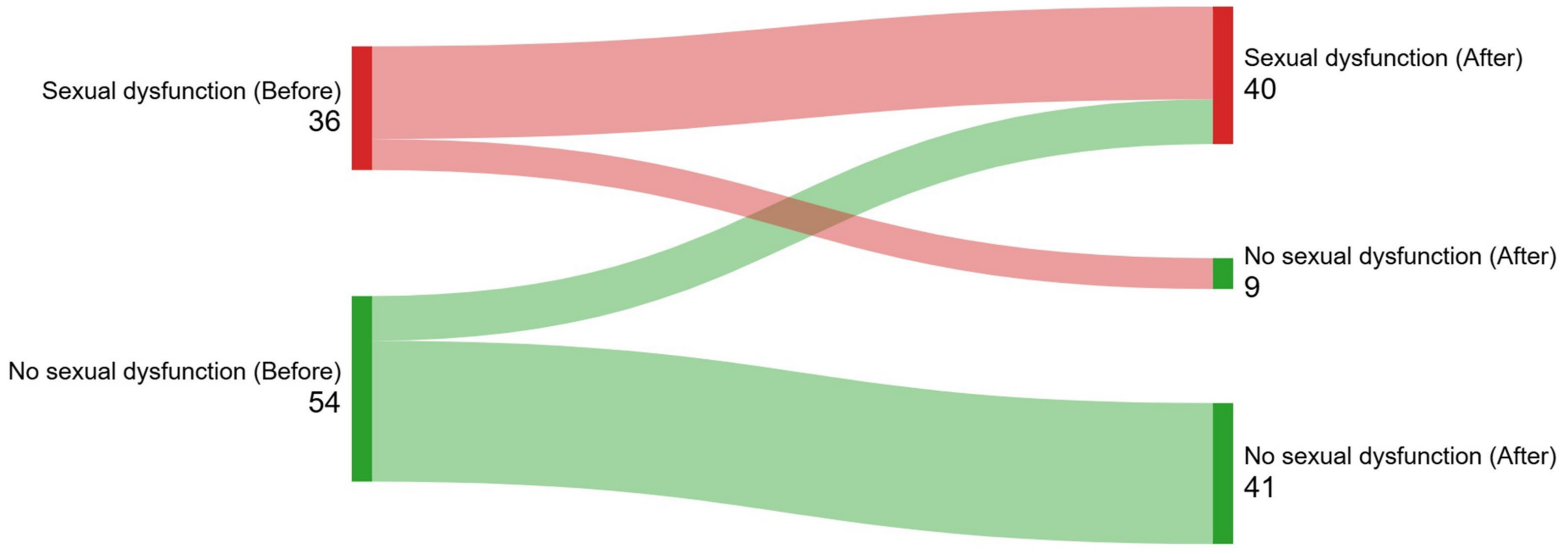
(Sankey diagram). Illustration of how participants’ sexual dysfunction status transitioned from the baseline measurement (before brachytherapy) to the second measurement at 3–6 months (after brachytherapy).

All patients who answered the questions regarding sexual function were sexually active. Sexual arousal and the ability to live without sex were the most frequently affected functions. There was no difference in the frequency of patients with dysfunctions before and after brachytherapy (*p* > 0.05) (Table 2). The incidence of sexual dysfunction (new cases after LDR brachytherapy) was 14.4%. Figure 2 (Sankey diagram) illustrates how participants’ sexual dysfunction status transitioned from the baseline measurement (before brachytherapy) to the second measurement at 3–6 months (after brachytherapy). Higher education was associated with a reduced risk of starting or maintaining sexual dysfunction following brachytherapy, independent of confounding factors (Table 3).

**Table 2:** Sexual function before and 3–6 months after LDR brachytherapy.

	Brachytherapy		*p*-value ^c^
Sexual function	Before (*n* = 139)	After (*n* = 90)	
Sexual satisfaction ^a^	23.0%	23.3%	0.954
Sexual desire has gone down ^b^	25.2%	26.7%	0.910
Sex enjoyment has gone down ^b^	22.3%	27.8%	0.617
Sexual arousal ^a^	38.8%	32.2%	0.208
Dyspareunia ^b^	13.7%	14.4%	0.874
Orgasms ^a^	30.9%	22.2%	0.110
Sexual difficulties with a partner, not when masturbating ^b^	5.0%	1.1%	0.194
Could live without sex (sexual interest) ^b^	31.7%	32.2%	0.916
Depressed and nervous because of sexual problems ^b^	5.0%	8.9%	0.458

a Never + almost never.

b always + almost always.

c Test on the difference between two proportions.

**Table 3:** Risk factors for sexual dysfunction 3–6 months after LDR brachytherapy.

Factor	Sexual dysfunction			
	No (moved to not having or remained without) (*n* = 50)	Yes (started or persisted after brachytherapy) (*n* = 40)	OR (95% CI)	*p*-value
Age, 60+	12.0%	20.0%	1.3 (0.6, 2.7)	0.523
Schooling				
Elementary	30.0%	45.0%	Reference	
Secondary	40.0%	35.0%	0.4 (0.1, 1.4)	0.149
High school	18.0%	15.0%	0.4 (0.1, 1.8)	0.267
Professional/postgraduate	**12.0%**	**5.0%**	**0.11 (0.01, 0.99)**	**0.049**
Major depression	16.0%	25.0%	0.9 (0.2, 4)	0.883
Moderate–severe somatic symptoms	16.0%	27.5%	3.8 (0.8, 19.1)	0.106
Comorbidity (any)	30.0%	32.5%	0.8 (0.3, 2.5)	0.754
Side effects/complications (any)	66.0%	67.5%	0.6 (0.2, 1.9)	0.385
Disease stage				
I	22.9%	15.0%	Reference	
II	25.0%	20.0%	1.6 (0.4, 6.9)	0.557
III	33.3%	37.5%	2.4 (0.5, 11.3)	0.258
IV	18.8%	27.5%	2.7 (0.5, 14.1)	0.238
Additional treatment				
Surgery and/or external beam radiation	22.0%	20.0%	Reference	
Chemotherapy + external beam radiation	48.0%	60.0%	1.5 (0.4, 6.2)	0.549
Surgery + chemotherapy + external beam radiation	30.0%	20.0%	0.6 (0.1, 2.9)	0.559

Bold indicated significant results (with *p* value < 0.05).

### Quality of life

**Figure 3:**
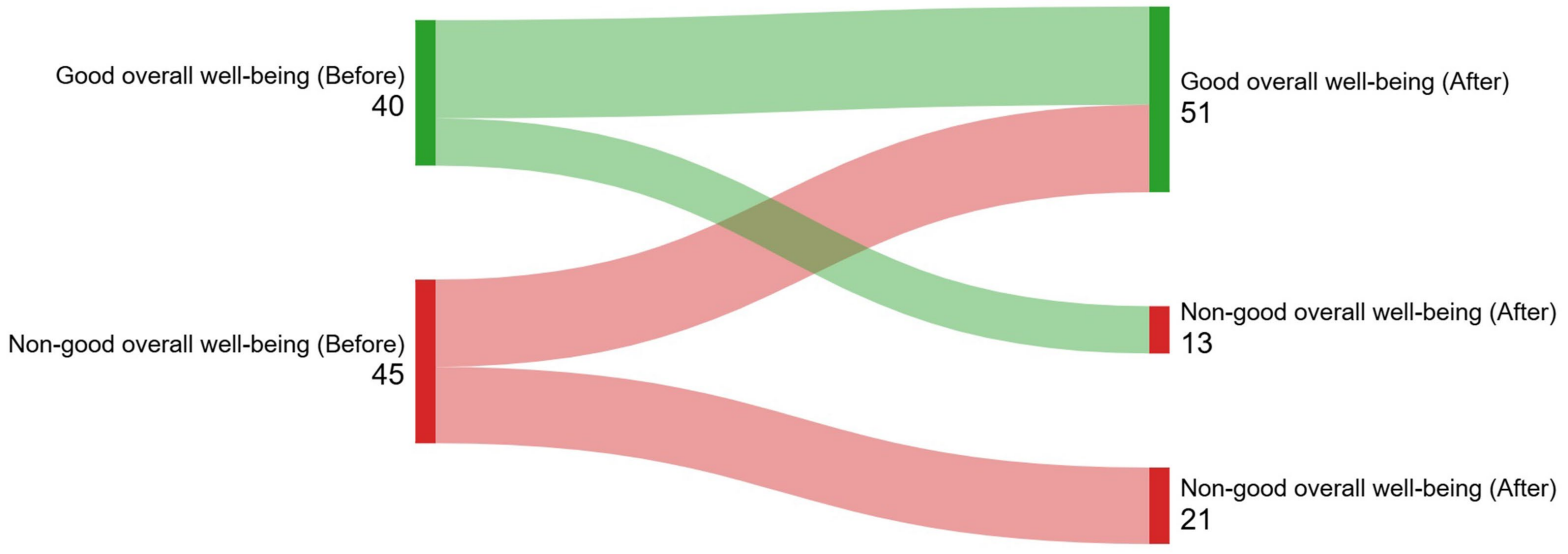
(Sankey diagram). Illustration of how participants’ overall wellbeing status transitioned from the baseline measurement (before brachytherapy) to the second measurement taken 3-6 months after brachytherapy.

**Figure 4:**
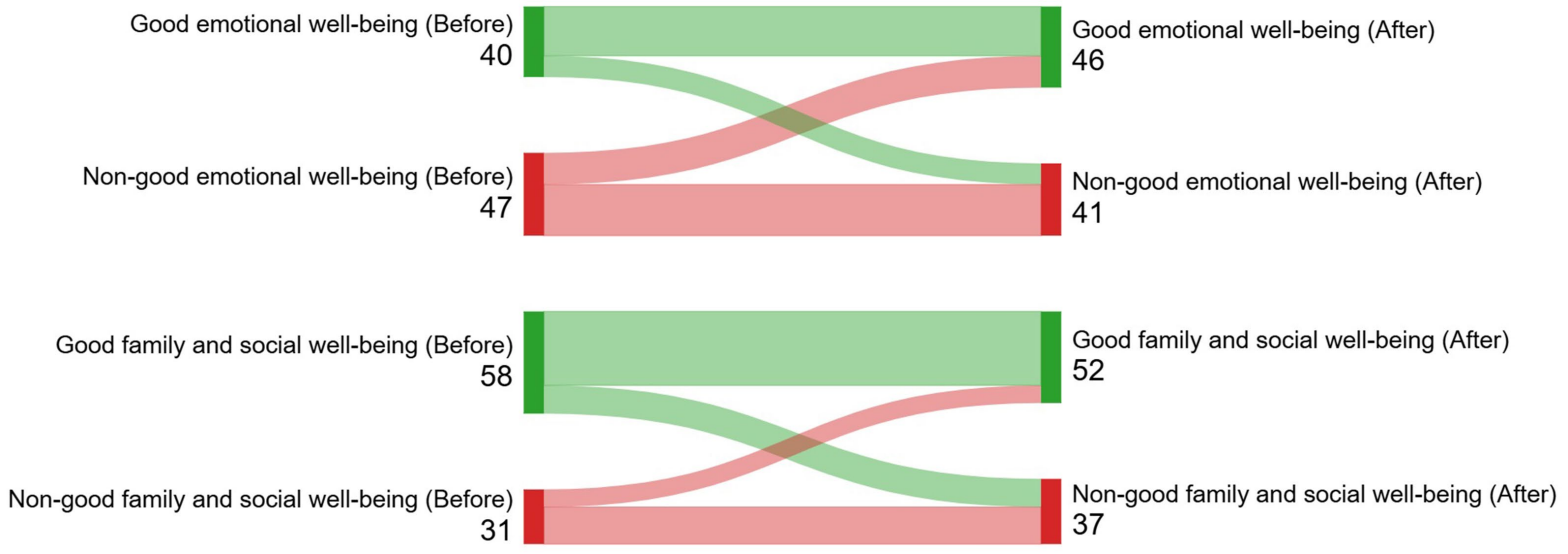
(Sankey diagram). Illustration of how participants’ emotional, family, and social wellbeing status transitioned from the baseline measurement (before brachytherapy) to the second measurement taken 3-6 months after brachytherapy.

**Figure 5:**
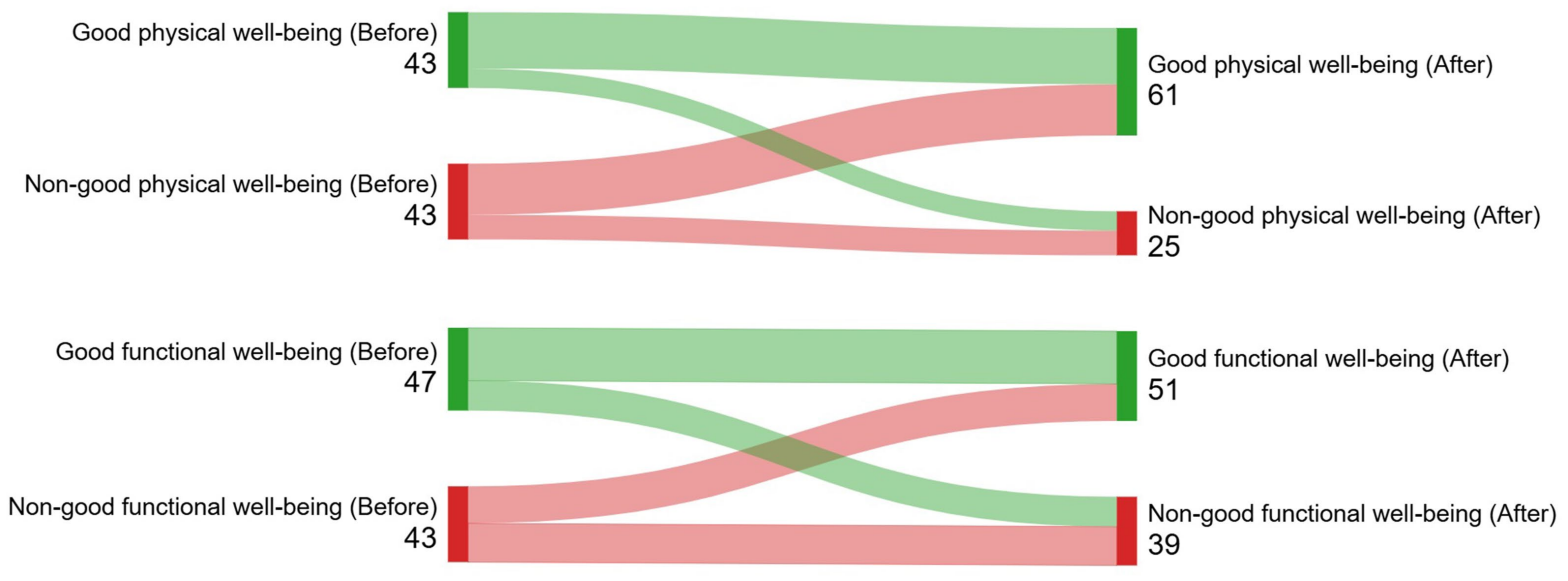
(Sankey diagram). Illustration of how participants’ physical and functional wellbeing status transitioned from the baseline measurement (before brachytherapy) to the second measurement taken 3-6 months after brachytherapy.

Physical wellbeing improved 3–6 months after brachytherapy (69.3 ± 24.1 vs. 78.7 ± 20.2, *p* < 0.001; effect size = 0.34). Emotional health was the most affected area, showing no improvement in wellbeing, remaining either poor or fair (Table 4). The incidence of good quality of life was 28.2% (new cases after LDR brachytherapy). Figures 3–5 (Sankey diagrams) illustrate the transition in participants’ overall and domain-specific quality of life statuses from the baseline measurement (before brachytherapy) to the second measurement taken 3–6 months after brachytherapy. Sexual dysfunction, major depression, and moderate–severe somatic symptoms reduced the likelihood of starting or maintaining a good quality of life after brachytherapy, independent of confounding factors (Table 5). Multivariate analysis showed only chemotherapy as a factor associated with a specific domain of quality of life: physical well-being (OR, 0.24; 95% CI, 0.06-0.94).

**Table 4:** Quality of life before and 3–6 months after LDR brachytherapy (n = 90).

	Brachytherapy		
Area	Before	After	*p*-values ^a^
Physical	69.3 ± 24.1	78.7 ± 20.2	0.008
Family and social wellbeing	78.1 ± 18.8	76.4 ± 18.0	0.390
Emotional wellbeing	67.1 ± 22.0	71.3 ± 21.2	0.137
Functional wellbeing	70.0 ± 21.1	73.7 ± 19.4	0.385
Overall wellbeing	71.3 ± 14.7	74.9 ± 13.8	0.069

a Wilcoxon signed-rank test for two related samples.

**Table 5:** Risk factors for good quality of life 3–6 months after LDR brachytherapy.

Factor	Quality of life			
	Non-good (moved to or persisted) (*n* = 34)	Good (started or maintained after brachytherapy) (*n* = 51)	OR (95% CI)	p-value
Age, 60+	17.6%	11.8%	0.8 (0.3, 2.1)	0.585
Schooling				
Elementary	47.1%	31.4%		
Secondary	35.3%	39.2%	1.1 (0.2, 5.4)	0.899
High school+	17.60%	29.40%	4.0 (0.6, 25.9)	0.148
Sexual dysfunction	**64.7%**	**31.4%**	**0.2 (0.1, 0.8)**	**0.019**
Major depression	**41.2%**	**2.0%**	**0.04 (0.004, 0.48)**	**0.011**
Moderate–severe somatic symptoms	**38.2%**	**5.9%**	**0.1 (0, 0.8)**	**0.032**
Comorbidity (any)	32.4%	31.4%	0.5 (0.1, 2.3)	0.373
Side effects/complications (any)	79.4%	62.7%	0.4 (0.1, 1.7)	0.199
Disease stage				
I	17.6%	20.4%		
II	17.6%	28.6%	1.1 (0.2, 6.7)	0.951
III	38.2%	28.6%	0.7 (0.1, 4.8)	0.681
IV	26.5%	22.4%	1.2 (0.2, 9.3)	0.844
Additional treatment				
Surgery and/or external beam radiation	23.5%	21.6%		
Chemotherapy + external beam radiation	58.8%	51.0%	1.9 (0.4, 9.8)	0.466
Surgery + chemotherapy + external beam radiation	17.6%	27.5%	3 (0.4, 23.3)	0.298

Bold indicated significant results (with *p* value < 0.05).

## Discussion

We estimated sexual dysfunction and quality of life before and after LDR brachytherapy in patients with cervical and endometrial cancer in a region where HDR brachytherapy units are not widely available. Sexual dysfunction was present in 44.4% of the study population (14.4% were new cases and 30% were prevalent cases). Roberts et al. (33) reported that 62.5% of endometrial and cervical cancer patients experienced at least one sexual difficulty after completing brachytherapy, chemotherapy, or external radiotherapy for more than 8 months. In the general population, female sexual problems affect up to 43% of women (34). Three areas of sexual dysfunction frequently overlap: interest/arousal, orgasmic, and genital-pelvic pain or penetration (35). We found that sexual interest and sexual arousal were the most frequent dysfunctions both before and after brachytherapy. Zomkowski et al. (19) reported a negative effect on lubrication (*p* = 0.05) and desire (*p* = 0.06) 7 days after completing HDR treatment. Kirchheiner et al. (20) documented less sexual activity than did a healthy population at baseline and 3 months after a combination of HDR, external beam radiotherapy, and chemotherapy. Studies have shown that patients with cervical and endometrial cancer experience more dysfunctions in terms of sexual arousal and orgasm intensity than healthy individuals do, along with entry dyspareunia (36, 37). Sexual dysfunction is a common issue among patients with cervical and endometrial cancer due to a combination of physical (fatigue and chronic pain), psychological (body image issues and fear of recurrence), and cultural factors (rights to enjoy a full sexual life, willingness to discuss sexual health concerns, and seek help). We have not yet studied how cervical and endometrial cancer relates to changes in body image and sexual dysfunction. Future studies should investigate this area further, as gynecological cancer treatments may lead to significant changes in body perception. Women may avoid or have less frequent sex because they fear that it could cause a recurrence of cancer or because they or their partners are concerned that cancer could be transmitted through sexual activity (38). We found that women with higher education levels had a lower risk of sexual dysfunction after brachytherapy, which is consistent with the findings of previous studies (39, 40). Women with higher levels of education often express more significant concern about their sexuality than women with lower levels of education do (41). Higher-educated women may feel more culturally empowered to discuss sexual issues openly. Access to care also contributes to the problem of sexual dysfunction due to the absence or insufficient availability of sexual health counseling or therapy services. Furthermore, women with cervical and endometrial cancer may not seek healthcare regarding their sexual function because they feel uncomfortable discussing their sex life with healthcare providers. Healthcare providers, for their part, may also feel uncomfortable talking about sexual health due to a lack of training, fear of offending or embarrassing the patient, underestimation of the incidence/prevalence of sexual dysfunction, and a lack of knowledge about the impact of sexual dysfunction on the wellbeing of patients with cervical and endometrial cancer. Patients with gynecologic cancer should receive counseling on how to cope with anticipated changes in sexual functioning. Addressing sexual dysfunction in patients with cervical and endometrial cancer requires a multidisciplinary approach, including medical treatment, psychological support, and counseling (34, 42).

Physical wellbeing significantly improved after LDR brachytherapy, with a difference of +9.4 compared to baseline. The physical domain is related to treatment side effects, which may negatively influence the quality of life. We found that the mean number of bothersome somatic symptoms at 3–6 months was lower than before, which may explain the improvement in physical wellbeing. Zomkowski et al. (19) reported no improvement in overall wellbeing 7 days after HDR brachytherapy but noted improvements in specific symptoms, including appetite, diarrhea, and constipation. Emotional health was the most affected area and remained unchanged, similar to previous reports (19, 20). Family and social wellbeing ranked high both before and after brachytherapy. This contrasts with the findings of Zomkowski et al. (19), who found that social wellbeing was the second most affected area, with no significant change between measurements. Kirchheiner et al. (20) reported that social functioning was low at baseline but improved, reaching a level comparable to that of the reference population within the first 6 months. These results underscore the importance of examining short-term quality-of-life effects across different populations to inform the planning of targeted interventions. The incidence of moderate–severe somatic symptoms and major depression was low (6.7%). Both reduced the likelihood of an overall good quality of life in addition to sexual dysfunction. Depression can exacerbate physical symptoms such as pain, fatigue, and sleep disturbances, severely impacting daily functioning and quality of life. It can overshadow positive experiences and reduce overall life satisfaction. Effective management of depression through therapy, medication, or a combination of both can significantly improve quality of life. It is crucial to conduct psychological assessments and gather a comprehensive medical history to identify women who may be at higher risk of developing depressive disorders during or after treatment. Strengthening social support networks and encouraging social engagement can also help mitigate the negative impact of depression. Sexual health in cancer patients is a crucial marker of quality of life, and information should be provided to patients about the sexual consequences of surgery. It is essential to identify which patients may suffer from sexual health issues to help improve their quality of life. Therefore, sexual function should be assessed regularly (34). Supportive care interventions assist patients in addressing sexual dysfunction issues and enhancing their wellbeing.

Any side effect may potentially impact sexual function and quality of life in women with gynecological cancer. We found that one out of two patients experienced a side effect, and gastrointestinal and genitourinary complications were frequent, occurring in 44 and 18% of patients, respectively. Chronic abdominal pain and cramping can make sexual activity uncomfortable or painful. Bloating, excessive gas, and bowel urgency may also cause physical discomfort during intimacy, especially when accompanied by urinary and anal incontinence complications. However, we did not observe a significant impact of these or other side effects on the patient’s sexual function and quality of life. Intensity, frequency, and side-effect management may have affected the results. Unfortunately, we did not have detailed information on the severity of the side effects. It would be interesting to continue this line of research to clarify the reasons for this lack of association. While the study focuses on the impact of LDR brachytherapy, we recognize that sexual function and quality of life may also be influenced by additional treatments, including surgery, chemotherapy, and external beam radiotherapy. Therefore, they were considered as control variables in the multivariate analysis, and the results for sexual function and quality of life were adjusted accordingly. We noticed no significant impact of additional treatments on the patient’s sexual function and overall quality of life as assessed by standardized questionnaires in this study. Chemotherapy was the only treatment associated with a reduction in physical wellbeing. Supportive care interventions can help patients manage chemotherapy side effects, thereby improving their overall wellbeing.

### Study limitations

Cultural factors such as shyness and embarrassment could have influenced sexual function responses, leading to the underestimation of sexual dysfunction rates. Feelings of shame can act as a bias in surveys of sexuality by influencing how participants perceive, recall, and report their sexual experiences, leading to misreporting. On the other hand, there is also the possibility that the sample was biased toward women who were more comfortable discussing sex, which affects the generalizability of the results. Additionally, we did not include a healthy comparison group, and it would be interesting to consider one in future studies. Several patients expressed interest in learning more about their treatment and its potential impact on sexual function. These patients received counseling from a health professional trained to guide and educate them on preventing or improving their sexual function. Expanding this area into a line of research to compare the impact of such health promotion would be valuable. The therapeutic regimens were heterogeneous; however, we were unable to analyze population subgroups, such as brachytherapy combined with radiotherapy vs. brachytherapy combined with surgery, due to the small sample sizes. The majority of patients lacked health insurance and belonged to an economically vulnerable social class; therefore, the results cannot be generalized to patients in middle- and high-socioeconomic statuses with private healthcare. Additionally, there is a recognized bias toward urban residents, as the majority of participants live in the metropolitan area of Monterrey, Mexico. Future studies should include residents of rural areas to evaluate whether the results differ based on area of residence.

## Conclusion

This study focused on a Mexican population from the northeast of the country. It was the first to assess sexual function and quality of life before and 3–6 months after LDR brachytherapy in a cohort of patients with cervical and endometrial cancer. More than 1 in 10 patients developed sexual dysfunction, and 3 in 10 experienced it before treatment. Physical wellbeing was the only quality of life that significantly improved after treatment. Higher education decreases the risk of starting or maintaining sexual dysfunction. Conversely, moderate–severe somatic symptoms, major depression, and sexual dysfunction adversely affect the ability to achieve or maintain a good quality of life after brachytherapy. Research in this area helps increase awareness and understanding of how healthcare providers can better support sexual and health-related wellbeing.

## Data Availability

The raw data supporting the conclusions of this article will be made available by the authors, without undue reservation.
