# Supplementary material for: Sexual dysfunction and quality of life in cervical and endometrial cancer patients before and after low-dose-rate brachytherapy: a cohort study
Source: Front Med (Lausanne). 2025 Apr 28;12:1584141. doi: 10.3389/fmed.2025.1584141 (PMC12066297; doi:10.3389/fmed.2025.1584141)
Supplement: Supplementary file 1 [file Data_Sheet_1.pdf]

Supplementary File 1. Classification of the change in status between the baseline measurement (before brachytherapy) and the second measurement taken 3-6 months after brachytherapy.

LDR brachytherapy		Outcome	Outcome
Before	After	(4 categories)	(2 categories)
**Sexual dysfunction categorization** [a]			
No	Yes	Changed from not having to have	Sexual dysfunction started or persisted after brachytherapy
Yes	Yes	Remained with sexual dysfunction	
Yes	No	Changed from having to not having	Moved to not having or remained without sexual dysfunction after brachytherapy
No	No	Remained without sexual dysfunction	
**Quality of life categorization** [b]			
Good	Non-good (poor or fair)	Changed from good to non-good	Moved to or remained non-good (poor or fair) after brachytherapy
Non-good (poor or fair)	Non-good (poor or fair)	Remained non-good	
Non-good (poor or fair)	Good	Changed from non-good to good	Good quality of life started or maintained after brachytherapy
Good	Good	Remained good	
**Somatic symptoms severity categorization** [c]			
Minimal-mild	Moderate-severe	Changed from minimal-mild to moderate-severe	Moderate-severe somatic symptoms started or persisted after brachytherapy
Moderate-severe	Moderate-severe	Remained moderate-severe	
Moderate-severe	Minimal-mild	Changed from moderate-severe to minimal-mild	Moved to or remained with minimal-mild somatic symptoms after brachytherapy
Minimal-mild	Minimal-mild	Remained minimal-mild	
**Major depression categorization** [d]			
No	Yes	Changed from not having to have	Major depression started or persisted after brachytherapy
Yes	Yes	Remained with major depression	
Yes	No	Changed from having to not having	Moved to not having or remained without major depression
No	No	Remained without major depression	

[a] Sexual dysfunction: SyDSF-AP mean score ≥ 2 (equivalent to sometimes, often, almost always, and always. [b] Good quality of life: FACT-G mean score ≥75 (equivalent to quite a bit and very much). [c] Moderate-severe somatic symptoms: PHQ15 ≥ 10. [d] Major depression: PHQ9 ≥ 9.
